# Case Report: Dynamic susceptibility-informed therapy for bile leak-associated intra-abdominal *Stenotrophomonas maltophilia* infection

**DOI:** 10.3389/fmed.2026.1876900

**Published:** 2026-08-24

**Authors:** Zhipeng Yang, Bin Zhu, Jianfeng Chen

**Affiliations:** Department of General Surgery, Suzhou BenQ Medical Center, Suzhou, Jiangsu, China

**Keywords:** antibiotic resistance, combination susceptibility testing, dynamic susceptibility evolution, intra-abdominal infection, *Stenotrophomonas maltophilia*

## Abstract

Iatrogenic bile leak complicated by intra-abdominal infection is a rare but serious complication after laparoscopic cholecystectomy. *Stenotrophomonas maltophilia* is intrinsically resistant to carbapenems and is an uncommon but clinically relevant pathogen of intra-abdominal infection, particularly in patients with immunosuppression, indwelling drains, and broad-spectrum antibiotic exposure. We report the case of a 55-year-old immunosuppressed man who developed bile leak due to Hem-o-lok clip-associated common bile duct injury after cholecystectomy. The clinical course is described by hospital day (HD) to align the manuscript with the treatment timeline. An initial culture from ascitic fluid indicated the presence of Gram-positive bacilli, whereas subsequent repeated cultures from abdominal drainage fluid yielded *S. maltophilia*, with no other bacterial species reported and negative anaerobic cultures. The isolate was initially resistant to levofloxacin and trimethoprim-sulfamethoxazole (TMP-SMX). An exploratory disk approximation test showed enlargement of the inhibition zone where the CZA and ATM disks met. This suggested a possible *in vitro* interaction. Because routine treatment options were limited, CZA plus ATM was administered together with renewed drainage and irrigation and was associated with clinical improvement. Repeat susceptibility testing revealed persistent levofloxacin resistance and susceptibility to TMP-SMX and minocycline, allowing step-down to oral minocycline 100 mg every 12 h after achieving source control. Antibiotics were stopped on HD 85, and the patient was discharged on HD 90 without recurrent fever, drainage, or imaging evidence of a recurrent collection during the final inpatient observation period. This case highlights the importance of repeated cultures, dynamic susceptibility reassessment, and source control in managing bile leak-associated intra-abdominal infection with repeated isolation of *S. maltophilia*.

## Introduction

Laparoscopic cholecystectomy is the gold standard for treating benign gallbladder diseases (1). A recent population-based cohort study found that bile leakage occurred in 1,738 of 152,413 patients after cholecystectomy, resulting in an incidence of 1.14% (2). However, complicated intra-abdominal infection secondary to iatrogenic bile leak remains a challenge, particularly when delayed recognition, repeated drainage, broad-spectrum antibiotic exposure, and impaired host defense coexist (3, 4).

*Stenotrophomonas maltophilia* (*S. maltophilia*) is an aerobic, non-fermenting Gram-negative bacillus found commonly in water and soil. Due to its strong adhesion and biofilm-forming ability, it can colonize medical devices and damaged tissue surfaces (5). *S. maltophilia* is an opportunistic pathogen, posing a higher risk to patients with malignancy, immunosuppression, prolonged hospitalization, or previous antimicrobial exposure (6, 7). Most clinically significant *S. maltophilia* infections affect the respiratory tract or bloodstream, while intra-abdominal infections are uncommon. In the Chinese Study for Monitoring Antimicrobial Resistance Trends, *S. maltophilia* accounted for 50 out of 3,420 aerobic and facultatively anaerobic Gram-negative isolates recovered from intra-abdominal specimens, representing 1.5% of the isolates (8). In a series of 110 *S. maltophilia* intra-abdominal infections, 47 were biliary tract infections, 84 were polymicrobial infections, and 104 patients had received antibiotics before the onset of infections, representing 42.7, 76.4, and 94.5%, respectively (9).

Broad-spectrum antibiotics are often required for treating intra-abdominal infection after bile leakage. However, prolonged exposure may suppress susceptible flora and allow intrinsically resistant organisms such as *S. maltophilia* to persist (5, 9). Current evidence remains limited regarding dynamic susceptibility changes and treatment strategies informed by patient-specific combination testing for bile leak-associated complicated intra-abdominal infection. This report describes a case of bile leak-associated intra-abdominal infection with repeated isolation of *S. maltophilia* in an immunosuppressed patient. The case emphasizes repeated source control, dynamic susceptibility monitoring, and cautious interpretation of combination susceptibility results.

## Case description

A 55-year-old man was admitted with right upper abdominal pain for 1 week. His medical history included thymoma resection and long-term oral cyclosporine treatment at 50 mg daily. Imaging upon admission showed cholecystolithiasis with cholecystitis, and laparoscopic cholecystectomy was performed on hospital day (HD) 2. Cefonicid (1 g daily) was administered as perioperative prophylaxis. Since the treatment timeline and source data were recorded by hospital day, the following case description uses HD rather than postoperative day.

On HD 3, the patient developed sudden severe abdominal pain, and the white blood cell (WBC) count increased to 21.1 × 10^9^/L. Imaging suggested bile leakage, and emergency laparoscopic exploration identified common bile duct erosion and leakage caused by Hem-o-lok clip compression. Laparoscopic repair and abdominal drainage were performed. Septic shock occurred postoperatively but resolved after fluid resuscitation and vasopressor support. Antibiotic therapy was initially escalated to ceftizoxime and subsequently to cefoperazone/sulbactam. Due to the persistence of systemic inflammation, the therapy was escalated to meropenem from HD 8 to HD 14. The first ascitic fluid culture after bile leak repair showed Gram-positive bacilli. No anaerobic bacteria were recovered in the anaerobic culture. Drainage and imaging surveillance continued.

On HD 22, *S. maltophilia* was first isolated from abdominal drainage fluid. The isolate was resistant to levofloxacin and trimethoprim-sulfamethoxazole (TMP-SMX). On HD 23, the patient was afebrile, the WBC count normalized to 8.7 × 10^9^/L, and the drainage volume decreased to approximately 30 mL/day of clear fluid. As the findings were initially interpreted as possible drain colonization rather than an active infection, the drain was removed. However, the subsequent course suggested that residual local infection had not been controlled.

**Figure 1:**
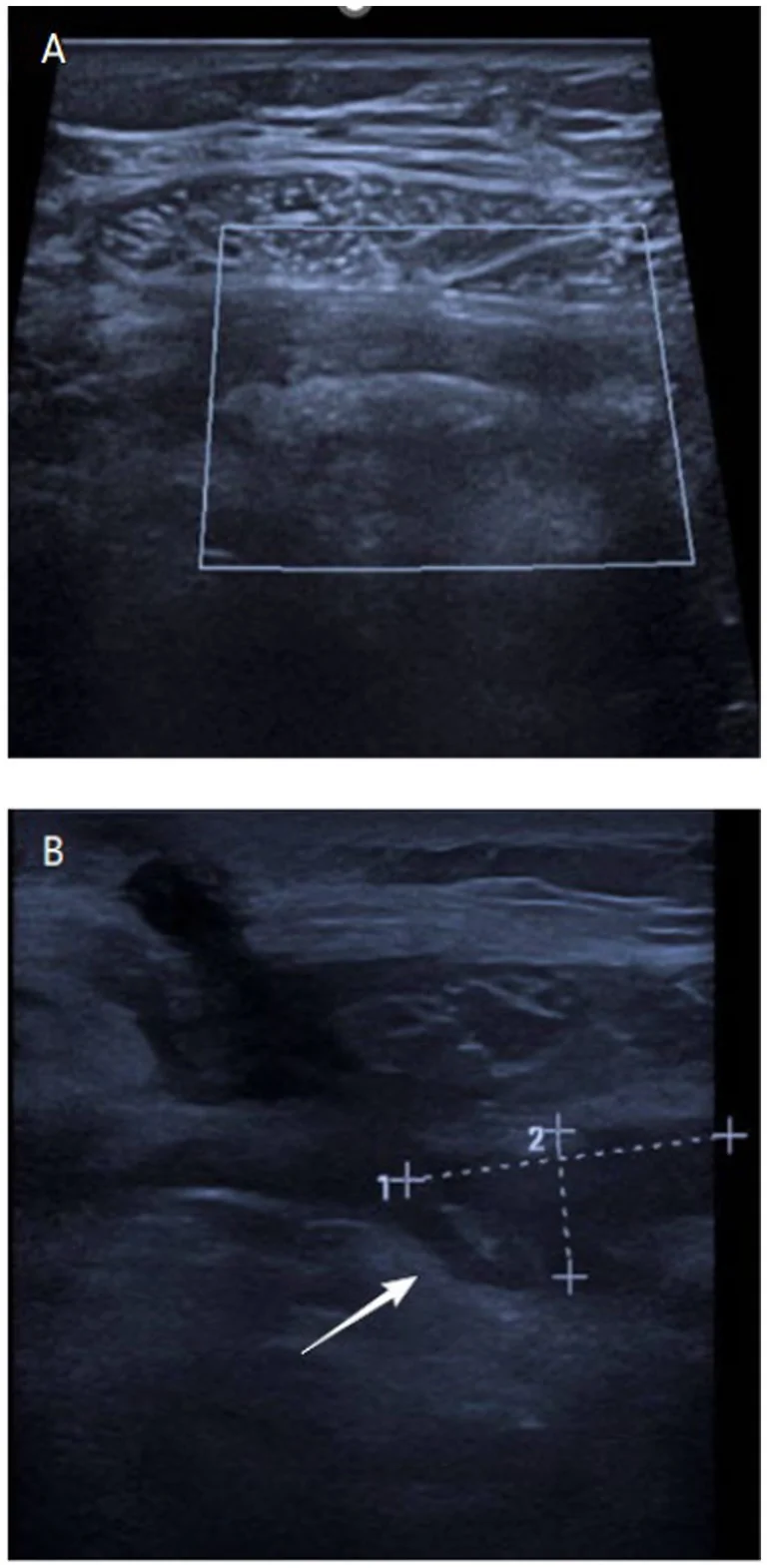
Ultrasound findings during recurrent abdominal collection. **(A)** On HD30, ultrasonography showed a 27 mm × 7 mm anechoic area with poor transparency and punctate echoes, consistent with localized fluid collection (white box). **(B)** On HD58, ultrasonography showed a 24 mm × 10 mm anechoic area with poor transparency and punctate echoes communicating with the abdominal wall sinus tract; flow sensation was observed with probe compression (white arrow).

**Figure 2:**
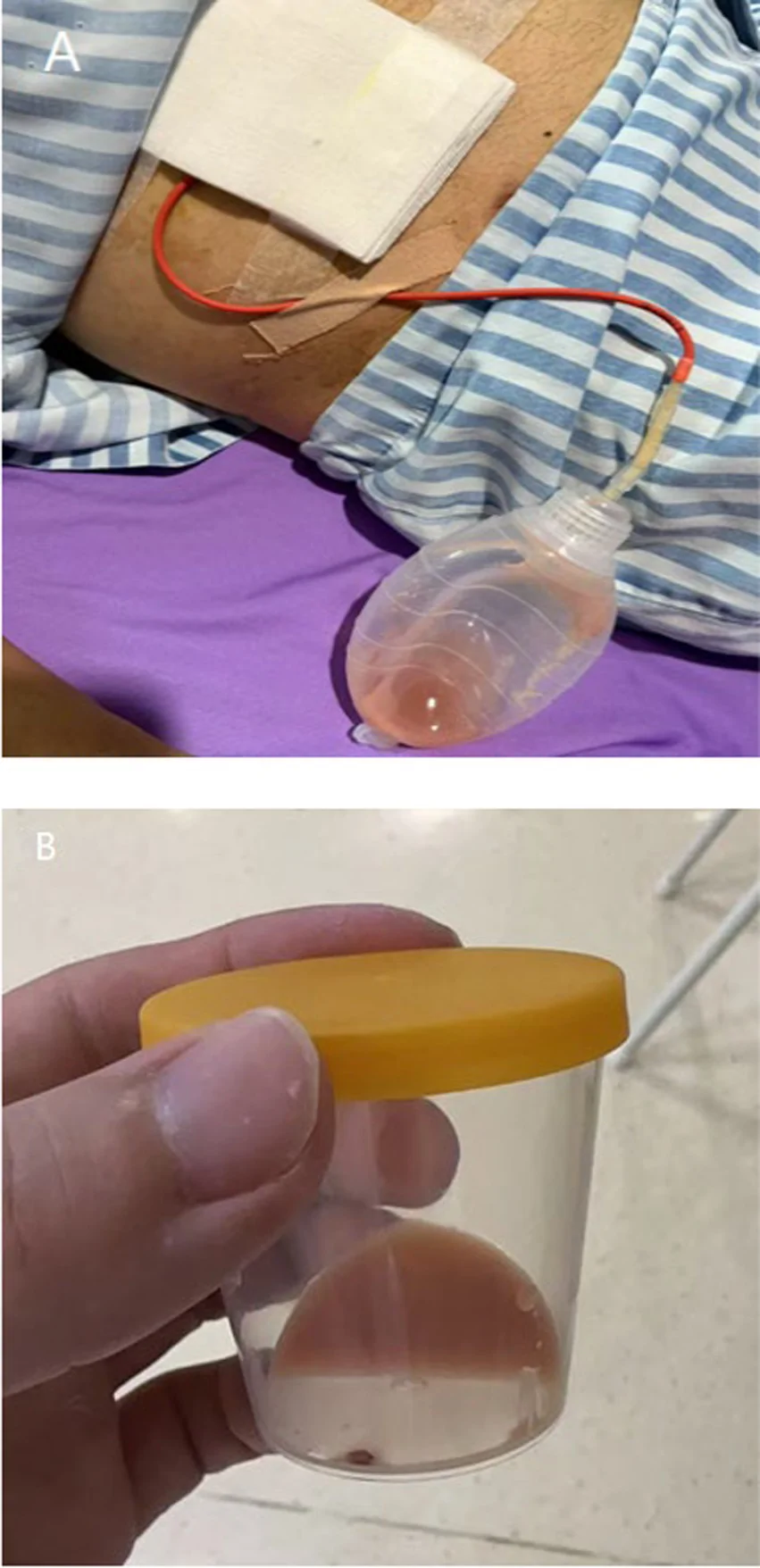
Ultrasound-guided source control on HD31. An 8 French (8Fr) drainage catheter was inserted through the original sinus tract and connected to a low-negative-pressure bulb, yielding thick dark-yellow fluid.

**Figure 3:**
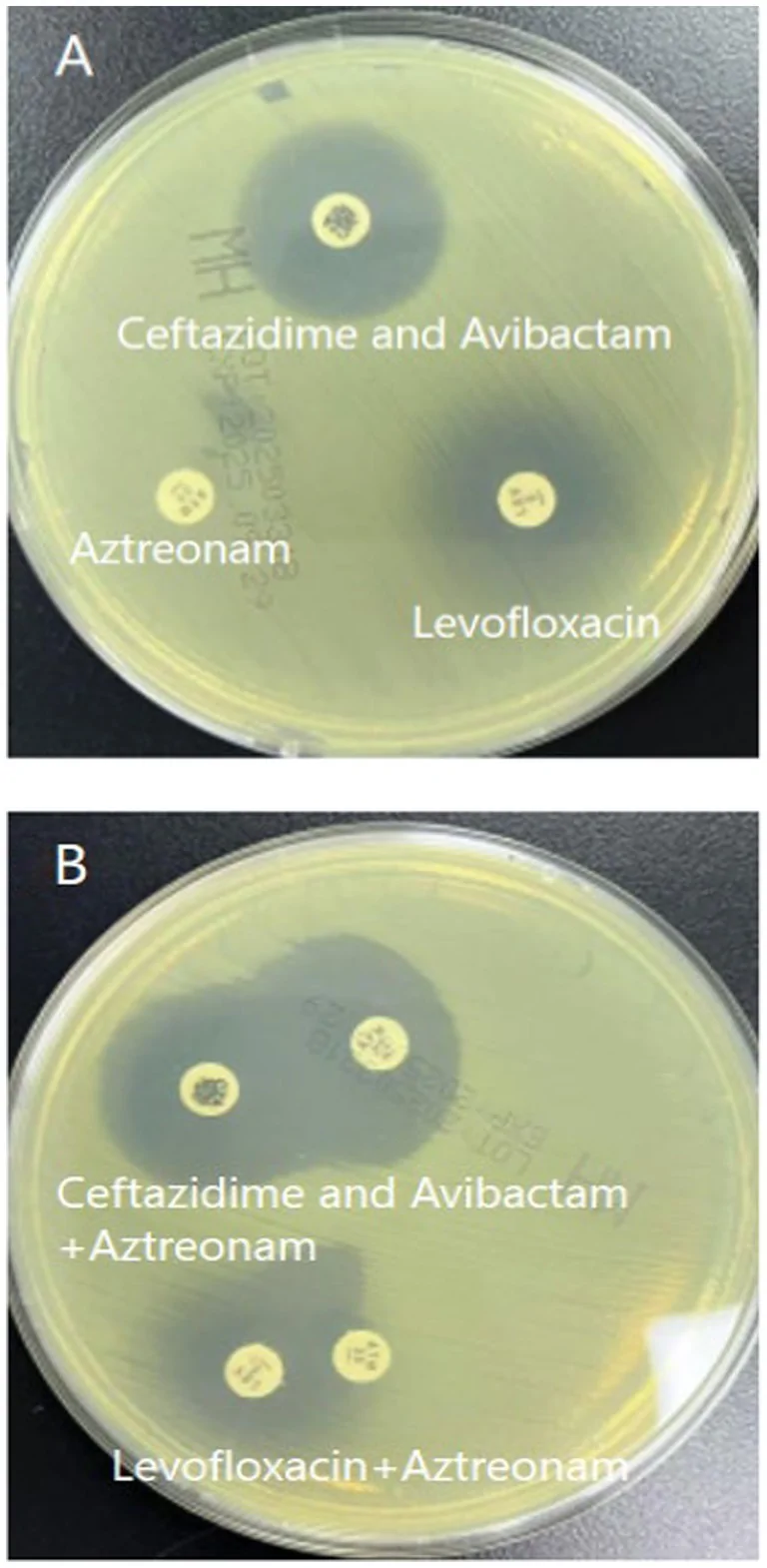
Disk diffusion and exploratory disk approximation testing on HD38. **(A)** Disk diffusion showed zone diameters of 11 mm for levofloxacin, 6 mm for aztreonam, and 23 mm for ceftazidime-avibactam. Levofloxacin was interpreted as resistant. CLSI provides no interpretive criteria for aztreonam or ceftazidime-avibactam against *S. maltophilia*. **(B)** Enlargement of the inhibition zone was observed where the CZA and ATM disks met. A similar finding was observed where the LEV and ATM disks met. These observations were reported descriptively.

**Figure 4:**
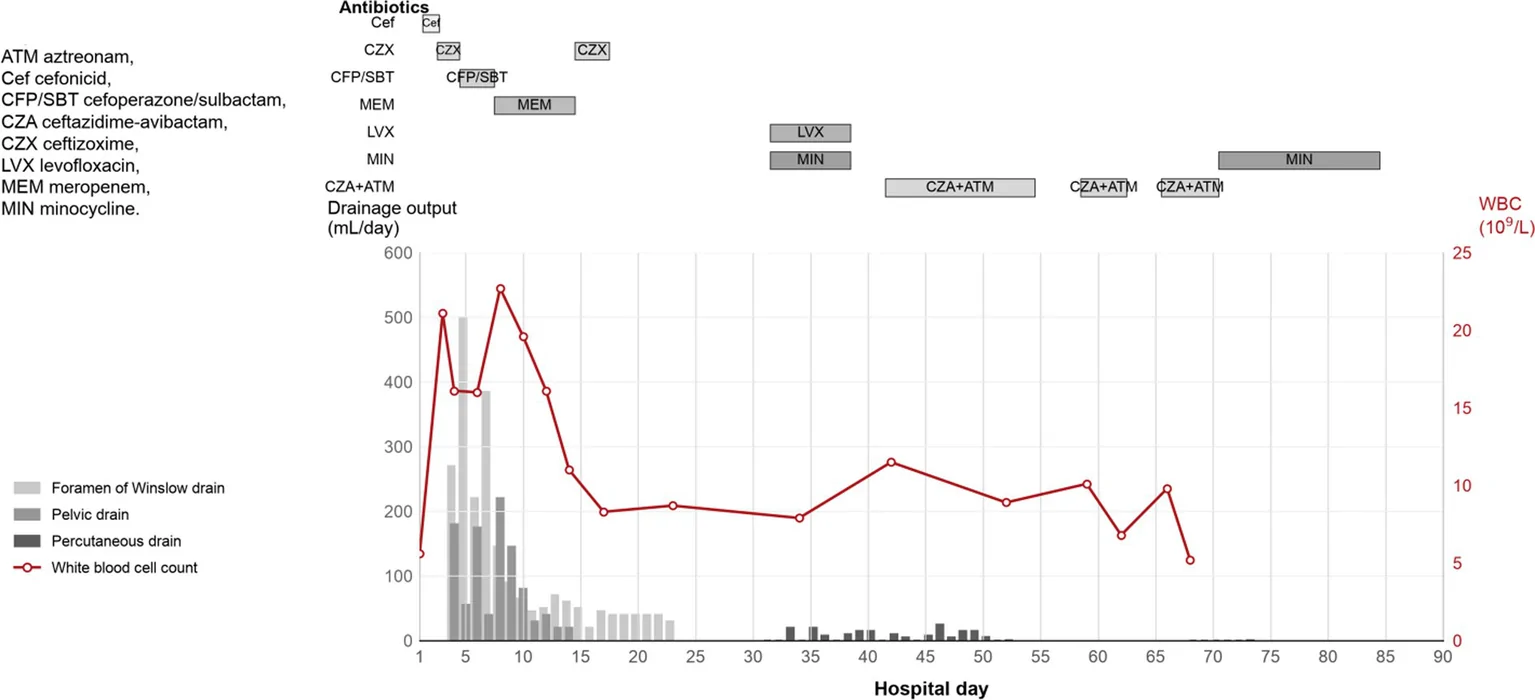
Integrated clinical course from HD1 to HD90. Bars show daily drainage output from the foramen of Winslow drain, pelvic drain, and percutaneous drain. The red line shows white blood cell count. Antibiotic exposure is shown above the graph by hospital day. The timeline summarizes bile leak repair on HD3, first isolation of *S. maltophilia* on HD22, the exploratory CZA and ATM disk approximation test on HD38, repeat susceptibility testing on HD70, percutaneous drain removal on HD73, antibiotic discontinuation on HD85, and discharge on HD90. ATM, aztreonam; Cef, cefonicid; CFP/SBT, cefoperazone/sulbactam; CZA, ceftazidime-avibactam; CZX, ceftizoxime; LVX, levofloxacin; MEM, meropenem; MIN, minocycline; WBC, white blood cell count.

The subsequent course was characterized by recurrent intra-abdominal collections requiring repeated drainage, irrigation, microbiologic reassessment, and antimicrobial adjustments. On HD 30, purulent discharge appeared at the original drain site, and an ultrasound scan showed a localized right mid-abdominal fluid collection (Figure 1A). Ultrasound-guided drainage yielded thick dark-yellow fluid, and an 8-Fr drainage catheter was left in place through the sinus tract on HD 31 (Figure 2). The same organism was recovered, and combination susceptibility testing was requested (Figure 3). Daily irrigation and drain management were performed. On HD 38, the exploratory disk approximation test showed marked enlargement of the inhibition zone where the ceftazidime-avibactam (CZA) and aztreonam (ATM) disks intersected (Table 1; Figure 3). CZA (2.5 g every 8 h) and ATM (2 g every 8 h) were administered from HD 42 to HD 54 in association with drainage control, clinical improvement, and gradual reduction in drainage output. The overall clinical course is summarized in Figure 4.

**Table 1:** Single-agent disk diffusion and exploratory disk approximation results.

Antibacterial agent	Breakpoint (mm)			Test result
	S	I	R	
Ceftazidime-avibactam	/	/	/	23 mm
Aztreonam	/	/	/	6 mm
Levofloxacin	≥17	14–16	≤13	11 mm, R
Ceftazidime-avibactam + aztreonam	Enlargement of the inhibition zone where the disks met			
Levofloxacin + aztreonam	Enlargement of the inhibition zone where the disks met			

Disk diffusion was used. Levofloxacin was interpreted using CLSI zone diameter criteria, with S ≥17 mm, I 14–16 mm, and R ≤13 mm. CLSI provides no interpretive criteria for ceftazidime-avibactam or aztreonam against *S. maltophilia*. Findings from the exploratory disk approximation test are reported descriptively. S, susceptible; I, intermediate; R, resistant.

A further local recurrence occurred after the drain was removed. *S. maltophilia* was isolated again on HD 55, and a low-grade fever with a recurrent localized collection that communicated with the abdominal wall sinus tract was identified on HD 58 (Figure 1B). Drainage was re-established, and daily irrigation was resumed. CZA and ATM were restarted from HD 59 to HD 70 while repeat culture and susceptibility testing were performed. On HD 70, repeat susceptibility testing reported persistent levofloxacin resistance, with an inhibition zone of 9 mm, and susceptibility to TMP-SMX and minocycline, with inhibition zones of 19 mm and 27 mm, respectively. As a result, oral minocycline (100 mg every 12 h) was prescribed as step-down consolidation therapy from HD 71 to HD 84 after drainage control. The drainage catheter was removed on HD 73 when output had decreased to 1 mL/day. Antibiotics were stopped on HD 85, and the patient remained clinically stable during the antibiotic-free observation period. He was discharged on HD 90 without recurrent fever, abdominal drainage, or imaging evidence of recurrent collection during the final inpatient observation period.

## Microbiological testing

Abdominal fluid and drainage fluid specimens were submitted for routine culture and organism identification through the hospital’s clinical microbiology workflow. Retrospective reports available to the authors identified *S. maltophilia* in subsequent drainage fluid cultures but did not specify the organism identification platform. According to hospital laboratory records for the relevant period, species-level identification was performed using the VITEK 2 system (bioMérieux, Marcy-l’Étoile, France). No molecular or genomic confirmation was performed. Single-agent susceptibility testing in the available reports was performed by disk diffusion using the antimicrobial agents included in the laboratory’s routine clinical panel at the time. The results were interpreted according to Clinical and Laboratory Standards Institute (CLSI) criteria when zone-diameter breakpoints were available. The susceptibility categories were transcribed from the contemporaneous clinical laboratory reports. The available retrospective records did not document the CLSI edition used. The available reports included levofloxacin and TMP-SMX for the initial isolate. The later report included levofloxacin, TMP-SMX, and minocycline. However, the CLSI did not provide single-agent interpretive criteria for CZA or ATM against *S. maltophilia*. Their zone diameters were reported for reference only. The routine reports did not include all agents listed by CLSI for *S. maltophilia*. Minimum inhibitory concentrations (MICs) were not determined because single-agent susceptibility testing followed the laboratory’s routine disk diffusion workflow. The isolates were not retained, so subsequent reference broth microdilution or additional susceptibility testing could not be performed. For the CZA-ATM combination, the laboratory conducted an exploratory disk approximation test. The returned report and plate images showed marked enlargement of the inhibition zone where the CZA and ATM disks intersected. The test used a 0.5 McFarland inoculum on Mueller–Hinton agar. Manufacturer details for the Mueller-Hinton agar and antimicrobial disks were unavailable in the retained laboratory records. The plates were incubated at 35 ± 2 °C in ambient air for 18–24 h. The disks contained ceftazidime-avibactam 30/20 μg, aztreonam 30 μg, and levofloxacin 5 μg. The CZA and ATM disks were placed 28 mm apart (center to center), and the ATM and levofloxacin disks were placed 10 mm apart (center to center). The inhibition zones were visually examined for enlargement where the disks intersected. No standardized interpretive criteria were applied. The result was not confirmed through MIC-based testing, checkerboard testing, time-kill testing, or the CLSI broth disk elution method. It was treated as an exploratory observation. Each clinical isolate was tested once. Technical triplicates were not performed.

## Discussion

*S. maltophilia* is a Gram-negative opportunistic pathogen of growing concern. It frequently affects patients with malignancy, immunodeficiency, prolonged antibiotic exposure, or long-term hospitalization and has been associated with poor outcomes (10–12). The majority of infections involve the respiratory tract and bloodstream, while intra-abdominal infections are uncommon (9). Enterobacterales are predominant bacteria in intra-abdominal infections. *S. maltophilia* is more often linked to environmental and healthcare reservoirs, although gastrointestinal colonization has been reported. This may partly explain its infrequent recovery from biliary specimens (5, 8, 9). Our patient had undergone thymoma resection and was receiving long-term cyclosporine. Biliary injury, indwelling drains, and repeated broad-spectrum antibiotic therapy were also present during the subsequent course.

The microbiological interpretation of this case requires caution. The initial ascitic fluid culture reported Gram-positive bacilli, indicating that an early polymicrobial intra-abdominal infection cannot be completely excluded. However, subsequent repeated cultures from the abdominal drainage fluid yielded *S. maltophilia*, with no other bacterial species reported and anaerobic cultures returning negative results. Therefore, this case is best described as bile leak-associated intra-abdominal infection with repeated isolation of clinically relevant *S. maltophilia* rather than a definitively monomicrobial infection. CZA combined with ATM is also active against some other Gram-negative organisms, so clinical improvement cannot be attributed solely to anti-*S. maltophilia* activity. Nevertheless, the repeated recovery of *S. maltophilia* from the recurrent drainage fluid, the timing of local relapse, and the response to renewed source control support its clinical relevance in the later course.

In this case, the recurrence of infection occurred after the early removal of the drain. From HD 22 to HD 23, the systemic signs improved, and the drainage output was low; however, local infection soon became evident through purulent drainage and a new collection. *S. maltophilia* can form biofilms and persist on medical devices and damaged tissue surfaces. Its intrinsic resistance may also favor persistence after prolonged exposure to broad-spectrum antibiotics (5, 13–15). Although biofilm formation was not directly tested, the repeated recurrence from the same drainage tract suggests that decisions regarding drain management should not rely only on fever, WBC count, or output volume. Imaging-confirmed source control may be especially important for immunosuppressed patients (16).

Treatment is difficult because *S. maltophilia* produces chromosomally encoded L1 metallo-*β*-lactamase and L2 serine β-lactamase, and it can also use efflux pumps, undergo permeability changes, possess mobile resistance determinants, and exhibit biofilm-associated tolerance (13, 14, 17–19). These mechanisms explain intrinsic carbapenem resistance and the limited reliability of many β-lactams. TMP-SMX has traditionally been considered a preferred agent, while levofloxacin and minocycline are commonly used alternatives; however, resistance, toxicity, and uncertain pharmacodynamic targets complicate management (20–23). In our patient, initial resistance to both TMP-SMX and levofloxacin made standard therapy difficult, prompting combination susceptibility testing.

The initial isolate was reported as resistant to TMP-SMX and levofloxacin. CZA plus ATM was selected because few routine options remained, and the combination was biologically plausible against the L1 and L2 *β*-lactamases of *S. maltophilia*. ATM is not hydrolyzed by L1. Avibactam inhibits L2 and may protect ATM (23, 24). A recent *in vitro* study using broth microdilution and time-kill methods found activity of ATM plus CZA against *S. maltophilia* complex isolates. Activity varied among isolates, and clinical evidence remains limited (24). In our case, single-agent disk-diffusion results were interpreted using CLSI criteria where applicable. The result of the disk approximation test with CZA and ATM was reported descriptively. The test showed marked enlargement of the inhibition zone where the CZA and ATM disks met. This suggested a possible *in vitro* interaction. We considered this result together with renewed drainage, irrigation, and the clinical response. It did not show that the drug combination alone caused the improvement. Subsequent susceptibility testing reported susceptibility to TMP-SMX and minocycline, whereas levofloxacin resistance persisted. We did not compare the serial isolates using molecular methods. The reason for the later susceptibility pattern remains unknown. Phenotypic variation or sampling of different bacterial subpopulations from the same infected space may have contributed (13, 18, 25, 26). Although TMP-SMX was also reported as susceptible on repeat susceptibility testing, minocycline was selected as oral step-down consolidation therapy because the patient had achieved renewed source control and clinical stabilization, and the isolate was susceptible *in vitro*. This choice reflected a feasible susceptible oral option rather than evidence that minocycline was superior to TMP-SMX in this setting. Current Infectious Diseases Society of America (IDSA) guidance favors high-dose minocycline, typically as part of combination therapy, for moderate-to-severe *S. maltophilia* infection (23). The 100 mg every 12 h regimen was used as oral step-down consolidation therapy after drainage control and clinical stabilization, and it should not be extrapolated to the initial treatment of moderate-to-severe active *S. maltophilia* infection.

This case suggests that repeated recovery of *S. maltophilia* from recurrent intra-abdominal collections in an immunosuppressed patient should prompt reassessment of source control before it is dismissed as drain colonization.

## Conclusion

This case highlights the importance of repeated microbiological assessment, imaging-guided source control, and dynamic susceptibility reassessment in bile leak-associated intra-abdominal infection with repeated isolation of *S. maltophilia*. When routine treatment options are limited, patient-specific combination testing may provide supportive information; however, antimicrobial decisions should be interpreted together with drainage adequacy and clinical response.

## Limitations

As this is a single case report, our findings may not be generalizable. Data from the hospital and surgical wards on the prevalence of *S. maltophilia* and other non-fermenting Gram-negative bacilli and their susceptibility patterns were unavailable during the study period, which limited our understanding of the local epidemiological context. Species identification was based on the VITEK 2 system without molecular or genomic confirmation. Closely related members of the *S. maltophilia* complex, including *S. sepilia*, could not be excluded (27). The basis for the susceptibility pattern reported on repeat testing was not investigated. Biofilm testing and MIC determination by reference broth microdilution were not performed. A definitively monomicrobial *S. maltophilia* infection cannot be proven because the initial ascitic fluid culture reported Gram-positive bacilli and prior antimicrobial exposure may have affected subsequent culture results, although later cultures repeatedly yielded *S. maltophilia* and anaerobic cultures were negative. Enlargement of the inhibition zone was observed where the CZA and ATM disks met an enlarged inhibition zone at their point of contact. The disk approximation test was not validated using MIC-based methods, checkerboard testing, time-kill testing, or the CLSI broth disk elution method. Therefore, the result was interpreted only as a possible *in vitro* interaction. Finally, oral minocycline 100 mg every 12 h was used as step-down therapy after source control and clinical stabilization; this dose is lower than the high-dose regimen favored in recent IDSA guidance for moderate-to-severe *S. maltophilia* infection. Further studies are needed to validate these observations.

## Data Availability

The original contributions presented in the study are included in the article. Further inquiries can be directed to the corresponding author.
